# City-level GDP estimates for China under alternative pathways from 2020 to 2100

**DOI:** 10.1038/s41597-025-06031-3

**Published:** 2025-11-05

**Authors:** Jinjie Sun, Rui Wang, Jiachen Wang, Shangchen Zhang, Mingyu Lei, Mengzhen Zhao, Junming Zhu, Can Wang, Wenjia Cai

**Affiliations:** 1 Department of Earth System Science, Institute for Global Change Studies, Ministry of Education Ecological Field Station for East Asian Migratory Birds, Tsinghua University, Beijing, 100084 China; 2 State Key Joint Laboratory of Environment Simulation and Pollution Control (SKLESPC), School of Environment, Tsinghua University, Beijing, 100084 China; 3 School of Management, Beijing Institute of Technology, Beijing, China; 4 School of Global Governance, Beijing Institute of Technology, Beijing, China; 5 School of Public Policy and Management, Tsinghua University, Beijing, 100084 China

**Keywords:** Energy and society, Environmental social sciences

## Abstract

Cities are key drivers of economic progress and play a decisive role in global climate action. Cities’ gross domestic product (GDP) data serves as a critical tool for evaluating economic progress and also offers a window into broader well-being, such as healthcare, education, and infrastructure. However, city-level GDP projections remain absent in China. This study uses the Cobb-Douglas production model to develop city-level GDP from 2020 to 2100, accounting for China’s unique socio-economic conditions. The dataset is validated by comparing its results with historical data and other future GDP scenarios. We develop 27 scenarios by varying technology, fertility, and intercity interaction across three levels each, considering China’s two-child/three-child policy, regional collaborative development, western development strategies, and technological advancements like AI. Among these, the Labor-Constraint Pathway and BAU Pathway closely align with the SSP1 and SSP2 scenarios, respectively. These scenarios provide a more accurate representation of future city-level GDP dynamics in China.

## Background & Summary

Gross Domestic Product (GDP) data is a fundamental indicator for assessing the economic status and development level of countries and regions. It is essential not only for economic progress but also for broader well-being, as it serves as a key indicator of a region’s capacity to invest in critical areas such as equity, education, healthcare, and overall quality of life^1^. When combined with other data, GDP serves as the basis for calculating key secondary indicators such as per capita GDP, energy intensity, and carbon emission intensity. These additional metrics offer a more comprehensive understanding of the relationship between economic growth and sustainability^2–4^. They provide key insights for future policy decisions, reflecting strategies to address economic, social, and environmental challenges, ensuring that economic objectives align with long-term sustainability.

The existing medium- and long-term economic projections embedded within the framework of Shared Socioeconomic Pathways (SSPs)^5–7^, may be less applicable to China due to recent effort on comprehensive poverty reduction, two/three-child policy, and carbon neutrality, particularly inequality SSP4 and fossil-fuel-dominant SSP5^8,9^. China’s poverty reduction strategy demonstrates a distinctive approach to addressing regional disparities through coordinated institutional mechanisms. The East-West Pairing Assistance program has established structured regional partnerships between developed eastern provinces and underdeveloped western regions, facilitating resource redistribution and industrial capacity transfer^10,11^. Demographic restructuring policies, including the 2016 two-child policy and 2021 three-child policy, represent strategic responses to labor market challenges. While immediate impacts on fertility rates remain limited^12^, these measures aim to mitigate long-term structural pressures from population aging. Incorporating these elements is essential to ensure the projections are both accurate and relevant to China’s evolving economic and social landscape.

Additionally, existing GDP projections predominantly available at national or provincial levels^13^ and more recently at gridded scales^14^, exhibit a critical gap in city-level spatial resolution. This limitation presents two fundamental limitations. First, the lack of urban-scale economic data hinders the examination of intercity economic interactions and spatial spillover effects, which are crucial for understanding regional growth patterns and economic agglomeration dynamics. Without granular city-level projections, it becomes inherently difficult to model how interurban collaboration, resource flows, and spatial externalities influence regional GDP development trajectories. Second, as emphasized in recent urban studies, cities serve as fundamental units for policy implementation, economic development, and environmental governance, concentrate resources, economic and social activities. The spatial granularity of city-level economic data provides critical insights into local economic dynamics, resource allocation efficiency, and the spatial distribution of socioeconomic activities, which are essential for addressing contemporary challenges in urban sustainability and regional development^15,16^.

In response to the research gaps identified, this study develops city-level GDP data for China, incorporating a comprehensive view of actual socioeconomic development and policies, to provide a stronger foundation for practical action at the city scale. By collecting city-level fixed capital stock, labor input, and total factor productivity data, we extend the widely used Cobb-Douglas method to the urban level, enabling a more accurate estimation of socioeconomic development in cities. Additionally, our city-level GDP pathways account for crucial drivers of economic development—technological advancements, labor dynamics, and inter-city interactions—while proposing assumptions aligned with current policy directions and avoiding the limitations of less applicable SSP scenarios. We provide a publicly available GDP dataset covering 27 scenario combinations, offering valuable insights into potential GDP trends and supporting informed decision-making.

## Methods

### General calculation function

**Fig. 1:**
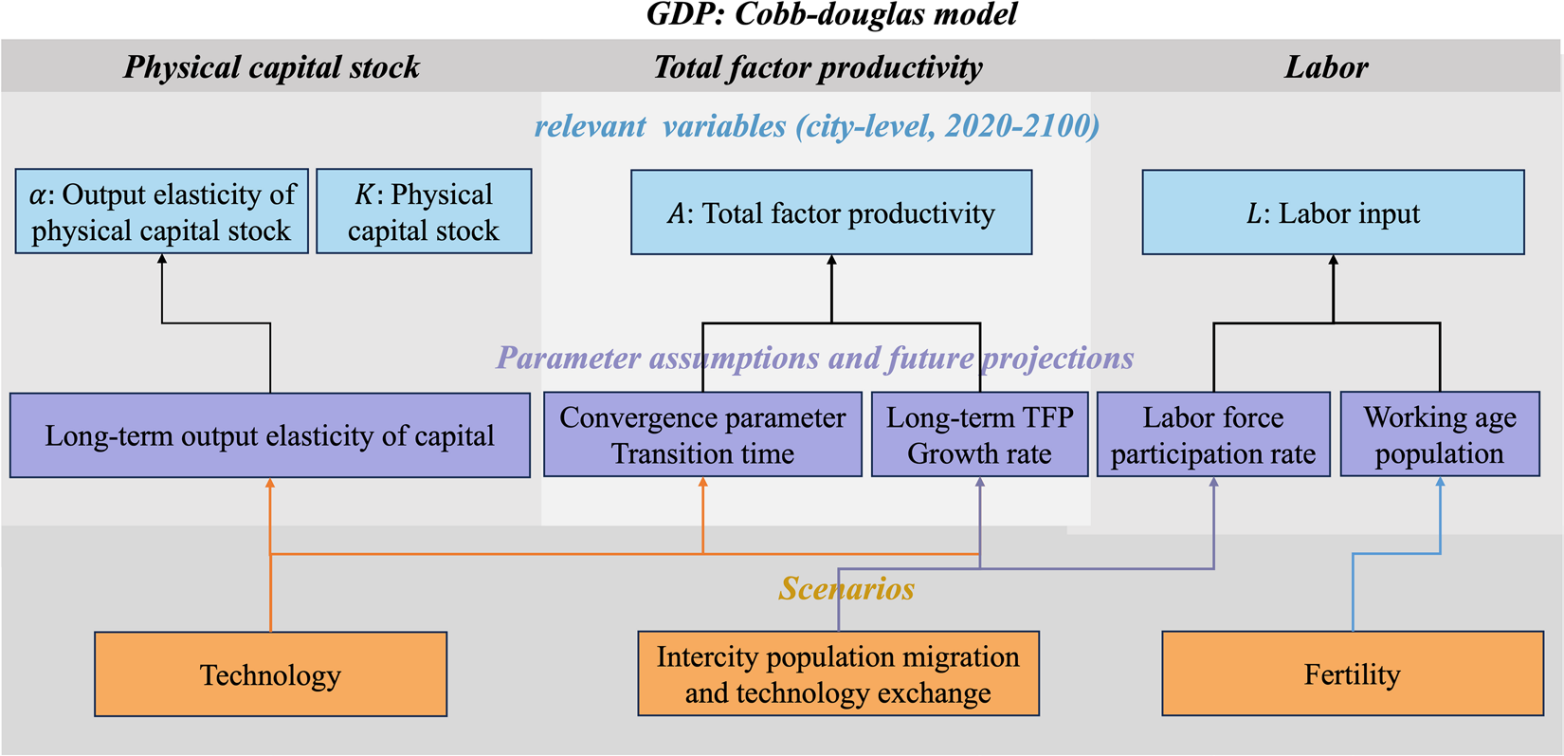
Framework of city-level GDP estimates in 2020–2100.

The framework of the GDP estimates is shown as Fig. 1. We adopt the widely used Cobb-Douglas production function^6,17,18^, driven by assumptions on long term trends of key economic growth drivers, including labor and capital input, and technical progress, to derive China’s city-level GDP projections under different pathways as shown below.

1 $${Y}_{t,c}={A}_{t,c}\times {{K}_{t,c}}^{{{\rm{\alpha }}}_{t,c}}\times {{L}_{t,c}}^{(1-{{\rm{\alpha }}}_{t,c})}$$

Where, *Y*_*t,c*_ represents GDP of city c in year t; *A*, *K*, *L* represent total factor productivity, physical capital stock, quality-adjusted labor input, respectively; *α* denotes output elasticity on capital.

Based on the Cobb-Douglas production function, the Solow growth model^19–21^ further explains the main driven of long-term economic growth. As diminishing marginal returns of capital accumulation serves as a short-term driver of growth, long-term growth relies on technological progress and improvements in human capital^22^. Therefore, we set technology development and fertility scenarios to effectively capture the crucial factors shaping the GDP growth trajectory. The fertility scenario determines future working-age population, thus affecting labor input. For the technology development scenario, technological progress affects both the long-term growth rate of total factor productivity across cities and the output elasticity of capital and labor.

The Cobb-Douglas model predicts that, the GDP gap among economies will narrow over the long term, allowing less developed economies to achieve faster growth through technology diffusion in open markets and the effective allocation of capital and labor^22^. Considering the finer-scale dynamics at city-level, we introduce the intercity interaction and disparities in the GDP projection, which has implications on migration and technology exchange. Migration among cities will determine the labor force distribution. Technology exchange among cities will influence the speed of city-level economic convergence.

### Historical data for long-term GDP projection

The model chooses 2020 as the reference year, based on city-level input dataset, assuring high-precision projection results in 2020–2100 at the city level. Due to the absence of city-level historical statistics, we first calculate city-level physical capital stock, output elasticity on capital, and total factor productivity in 2020. The input datasets used for the calculation can be found in Table 1. Provincial statistical yearbooks^23^ serve as the primary source, as they include detailed economic indicators for individual cities within each province.

**Table 1:** description of historical data input.

Variable	Data	variables	time	source
Physical capital stock, Capital output elasticity	Fixed capital investment	*I*	1952–2020	City-level data compiled from provincial statistical yearbooks
	Price index of fixed capital investment	*PI*	1952–2020	Provincial level data compiled from national statistical yearbooks
TFP, Capital output elasticity	GDP	*Y*	2020	City-level data compiled from provincial statistical yearbooks
TFP, Labor	Urban-rural differentiated labor statistics	*L*	2020	City-level data compiled from sixth nationwide population census
TFP growth rate	GDP index	*GY*	2020	City-level data compiled from provincial statistical yearbooks
	Growth rate of labor input	*GL*	2020	City-level data compiled from provincial statistical yearbooks

The city-level GDP and urban-rural differentiated labor statistics, are respectively obtained from provincial statistical yearbooks and sixth nationwide population census. Given that the Chinese government doesn’t publish physical capital stock figures, we adopt the widely used perpetual inventory approach^24,25^, compiling a historical dataset of fixed capital investment and its price index from 1952 to 2020, to calculate the physical capital stock in 2020^26,27^ as shown in Eq. (2).

2 $${K}_{t+1,c}={I}_{t+1,c}/{{PI}}_{t+1,c}+(1-\delta )\times {K}_{t,c}$$

3 $${{ln}{GK}}_{t,c}=\mathrm{ln}\frac{{K}_{t+1,c}}{{K}_{t,c}}$$

Where, *K*_*t,c*_ represents capital stock of city c in year t, which ranges from 1952 to 2019; *I*, *PI*, and *δ* denote physical capital investment, price index of physical capital investment, and the depreciation rate of capital, respectively. Then *GK*_*t,c*_, the growth rate of capital stock is calculated in Eq. (3).

4 $${{\rm{\alpha }}}_{2020,\mathrm{c7}}=p\cdot \frac{{K}_{2020,c}}{{Y}_{2020,c}}$$

Where, ${{\rm{\alpha }}}_{2020,c}$ represents capital output elasticity of city c in 2020; p denotes the price of capital, also the return rate on gross capital investments. Following empirical studies on regional capital productivity in China, we apply differentiated rates: 0.10 for western regions, 0.12 for central cities, and 0.17 for eastern coastal areas^28^. *K*, *Y* represent physical capital stock and GDP in 2020, respectively.

The total factor productivity (TFP) and TFP growth rate of the base year 2020 are required in the economic projection model. The TFP growth rate in 2020 is calculated via the Solow residual method as follows.

5 $${{GT}}_{2020,c}={{GY}}_{2020,c}-{{\rm{\alpha }}}_{2020,c}{{GK}}_{2020,c}-(1-{{\rm{\alpha }}}_{2020,c}){{GL}}_{2020,c}$$

Where, ${{GT}}_{2020,{c}}$, ${{GY}}_{2020,c}$, ${{GK}}_{2020,c},\,{{GL}}_{2020,c}$ represent the TFP growth rate, GDP growth, capital stock, labor growth rate of city c, respectively; α represents output elasticity of capital.

### The calculation of China’s GDP under different scenarios

#### Labor

The projections for the annual total urban population distinguishing different age groups are sourced from Zhang *et al*.^29^. The urban and rural structures and education level are based on provincial population estimates by Chen *et al*.^30^, due to the current lack of urban statistics. We project the long-term labor supply for urban and rural separately. The calculation method is described in Eqs. (6–8).

6 $${L}_{c,t}={\sum }_{{\rm{s}},{\rm{q}}}{{\rm{H}}}_{{\rm{s}},{\rm{c}},{\rm{t}}}\times {{\rm{LFPR}}}_{{\rm{s}},{\rm{q}},{\rm{c}},{\rm{t}}}\times {{\rm{WAP}}}_{{\rm{s}},{\rm{q}},{\rm{c}},{\rm{t}}}$$

Where, ${L}_{c,t}$ represents labor input of city c in year t; s and q represent the type of urban or rural and two age classes for working age people; H represents education level; LFPR and WAP represent labor force participation rate and working age population. Based on retirement age, working age people are categorized into two groups: 16–55 years and above 55 years for urban area; and 16–65 years and above 65 years for rural area, which is consistent across all cities.

7 $${{\rm{H}}}_{{\rm{s}},{\rm{c}},{\rm{t}}}=\left\{\begin{array}{cc}{{\rm{e}}}^{0.134\cdot {{\rm{MYS}}}_{{\rm{s}},{\rm{c}},{\rm{t}}}}, & {{\rm{MYS}}}_{{\rm{s}},{\rm{c}},{\rm{t}}}\le 4\\ {{\rm{e}}}^{[0.536+0.101\cdot ({{\rm{MYS}}}_{{\rm{s}},{\rm{c}},{\rm{t}}}-4)]}, & 4 < {{\rm{MYS}}}_{{\rm{s}},{\rm{c}},{\rm{t}}}\le 8\\ {{\rm{e}}}^{[0.94+0.068\cdot ({{\rm{MYS}}}_{{\rm{s}},{\rm{c}},{\rm{t}}}-8)]}, & {{\rm{MYS}}}_{{\rm{s}},{\rm{c}},{\rm{t}}} > 8\end{array}\right.$$

8 $${{\rm{MYS}}}_{{\rm{s}},{\rm{c}},{\rm{t}}}=\left({\sum }_{{\rm{edu}}}{{\rm{WAP}}}_{{\rm{s}},{\rm{q}},{\rm{c}},{\rm{t}},{\rm{edu}}}\times {{\rm{YoS}}}_{{\rm{edu}}}\right)/{\sum }_{{\rm{edu}}}{{\rm{WAP}}}_{{\rm{s}},{\rm{q}},{\rm{c}},{\rm{t}},{\rm{edu}}}$$

Where, ${{\rm{H}}}_{{\rm{s}},{\rm{c}},{\rm{t}}}$ stands for education level in city c in year t, s represents the distinction between rural and urban areas; MYS denotes mean years of schooling, assuming education acts as a scaling factor on labor according to the equation; ${{\rm{YoS}}}_{{\rm{edu}}}$ represents schooling years of population with different education level, edu symbolizes the seven stages of education in China, which are consistent with results in the census: illiterate, primary education, junior high school, senior high school, college education, bachelor’s education, master’s education and above, corresponding to 0, 6, 9, 12, 16, 16, and 16 years of schooling, respectively. Due to the absence of city-level datasets distinguishing urbanization and education level, we use provincial projection of Chen *et al*.^30^ to calculate the ${{\rm{H}}}_{{\rm{s}},{\rm{c}},{\rm{t}}}$ in cities.

In this study, we assume three fertility scenarios: low fertility with insufficient policy, stable fertility as the business-as-usual scenario, and high fertility assuming policy to be effective ideally, which is consistent with the assumptions of Zhang *et al*.^29^. Furthermore, the migration level also influences the labor input at city level, which is reflected in intercity interaction and disparities scenario. The migration rates from third- and fourth-tier cities to first- and second-tier cities is assumed gradually decreasing to zero after 2020, while HighInter holds the same migration level as 2010 before 2020; MedInter describe the business-as-usual situation before 2020; LowInter assumes higher migration rates leading to greater inequality between urban areas, which is consistent with Zhang *et al*.^29^.

China’s labor force participation rate is experiencing a high-level decline. Considering the current projections for the growth rate of China’s LFPR and the rates for major countries in 2021^31^—61.66% for the United States, 65.18% for Japan, and 60.55% for Germany—we assume a steady decline in the LFPR^32,33^ at a rate of −0.26% until it stabilizes in 2030 in the HighInter and MedInter scenario. In the context of intensified migration and a widening gap among urban areas, the saturation of the employment market in major cities, coupled with labor outflow from third- and fourth-tier cities, could hinder economic development and employment opportunities. Therefore, we hypothesize that in the LowInter scenario, the overall employment rate will decline more rapidly at a rate of −0.51% until it stabilizes in 2030.

#### Total factor productivity

The approach of TFP projection is based on two components: (I) TFP projection of technological leader, which is Shenzhen in this study, (II) TFP projection of other cities, which is transitioned from historically dominated to convergence-based long-term TFP growth.

9 $${{\rm{A}}}_{{\rm{L}},{\rm{t}}}={{\rm{A}}}_{{\rm{L}},2020}\cdot {\left\{1+\left[{{\rm{g}}}_{{\rm{L}}}+({{\rm{g}}}_{{\rm{L}},2020}-{{\rm{g}}}_{{\rm{L}}})\cdot {{\rm{e}}}^{-{\rm{\gamma }}{\rm{t}}}\right]\right\}}^{{\rm{t}}}$$

Where, A_L_ is the TFP of the technological leader in year t, A_L,2020_, g_L,2020_ is the TFP and TFP growth rate of the technological leader in base year 2020; $\gamma$ is the transition rate; g_L_ is long-term TFP growth rate, which is respectively 0.6% for LowTech, 0.8% for MedTech, 1.0% for HighTech per year^6^, to depict different level of technological advancement.

The TFP of all other cities is calculated based on the Eq. (10), which concludes two stages: short term dynamics based on empirically derived initial TFP growth rate, and convergence for catching-up with the technological leader in the long run.

10 $${{\rm{A}}}_{{\rm{c}},{\rm{t}}+1}=\left\{\begin{array}{c}\frac{{\rm{t}}\cdot \left\{{{\rm{A}}}_{{\rm{L}},{\rm{t}}+1}-\left[{{\rm{A}}}_{{\rm{L}},{\rm{t}}+1}-{{\rm{A}}}_{{\rm{c}},{\rm{t}}}\right]\cdot {{\rm{e}}}^{-{\rm{t}}\cdot {{\rm{\beta }}}_{{\rm{c}}}/10}\right\}+({{\rm{\tau }}}_{{\rm{c}}}-{\rm{t}})\cdot (1+{{\rm{g}}}_{{\rm{c}}})\cdot {{\rm{A}}}_{{\rm{c}},{\rm{t}}}}{{\rm{\tau }}({\rm{r}})},{\rm{t}}\le {{\rm{\tau }}}_{{\rm{c}}}\\ \max \left\{{{\rm{A}}}_{{\rm{c}},{\rm{t}}},{{\rm{A}}}_{{\rm{L}},{\rm{t}}+1}-\left[{{\rm{A}}}_{{\rm{L}},{\rm{t}}+1}-{{\rm{A}}}_{{\rm{c}},{\rm{t}}}\right]\cdot {{\rm{e}}}^{-{\rm{t}}\cdot {{\rm{\beta }}}_{{\rm{c}}}/10}\right\},{\rm{t}} > {{\rm{\tau }}}_{{\rm{c}}}\end{array}\right.$$

Where, ${{\rm{A}}}_{{\rm{c}},{\rm{t}}},{{\rm{A}}}_{{\rm{L}},{\rm{t}}}$ represent the TFP of city c and the technological leader in year t, respectively; ${{\rm{g}}}_{{\rm{c}}}$ represents the initial growth rate of city c, β, ${\tau }$ represent convergence parameter and the transition time between the two phases of TFP growth. The first equation represents the evolution of the TFP as a transition from the historically based evolution toward the convergence-based TFP, while the second formula describes the long-term convergence process.

To ensure that GDP growth predictions are realistic and reasonable, we differentiate the parameters for each city accordingly, assuming that the most advanced cities will experience comparatively fast convergence to the technology leader. Furthermore, the assumptions of technology development and intercity exchange will also shape the progress of convergence towards technological leader. Under the higher level of technological development, economy across cities is expected to converge on a higher level and therefore within a longer time horizon, with the setting of larger transition time. We also assume that technological exchange and balanced development between cities is described by convergence rate, which is 0.003–0.02 in MedInter scenario, while HighInter and LowInter scenario are respectively 50% higher and 50% lower than MedInter scenario.

#### Physical capital stock

In terms of physical capital stock, a recursive model is adopted to project future physical capital stocks.

11 $${{\rm{K}}}_{{\rm{c}},{\rm{t}}}={\left[\frac{{{\rm{K}}}_{{\rm{c}},{\rm{t}}-1}}{{{\rm{L}}}_{{\rm{c}},{\rm{t}}-1}}\right]}^{\frac{1-{\alpha }_{{\rm{c}},{\rm{t}}-1}}{1-{\alpha }_{{\rm{c}},{\rm{t}}}}}\cdot {\left[\frac{{\alpha }_{{\rm{c}},{\rm{t}}}}{{\alpha }_{{\rm{c}},{\rm{t}}-1}}\cdot \frac{{{\rm{A}}}_{{\rm{c}},{\rm{t}}}}{{{\rm{A}}}_{{\rm{c}},{\rm{t}}-1}}\right]}^{\frac{1}{1-{\alpha }_{{\rm{c}},{\rm{t}}}}}\cdot {{\rm{L}}}_{{\rm{c}},{\rm{t}}}$$

Where, ${{\rm{K}}}_{{\rm{c}},{\rm{t}}}$, ${{\rm{L}}}_{{\rm{c}},{\rm{t}}}$, ${\alpha }_{{\rm{c}},{\rm{t}}}$, ${{\rm{A}}}_{{\rm{c}},{\rm{t}}}$ represent physical capital stock, labor input, output elasticity of capital, and TFP of city c in year t, respectively. Future technological developments and the large-scale application of AI technologies are expected to bring capital investment contributing more significantly to GDP growth compared with labor input. Therefore, the long-term output elasticity of capital is assumed to be stable at the level of 0.3 for LowTech scenario, 0.35 for MedTech scenario, and 0.4 for HighTech scenario.

### Scenario assumptions

#### Technology advancement

This emphasizes the vital role of technological progress in GDP growth, illustrating that effective resource allocation and innovation are fundamental to economic development. In this study, we discuss the advancement of the technology in three levels, namely HighTech, MedTech, and LowTech. The LowTech scenario assumes that technology progresses slowly. The MedTech scenario represents moderate technological development, driving improvements in productivity and efficiency. The HighTech scenario envisions revolutionary transformations driven by technologies like AI and automation, resulting in enhanced innovation and improved resource allocation, ultimately fostering robust economic growth.

#### Fertility

China’s current stage of socioeconomic development has brought urban fertility rates into a new era. The government has introduced a range of fertility policies, including the two-child and three-child initiatives, aimed at promoting higher birth rates and stimulating economic growth. In this study, we establish three fertility rate scenarios depict potential population trends and the effectiveness of fertility policies, while LowFer, MedFer, and HighFer scenario respectively reflect insufficient, moderate and effective policy promoting.

#### Intercity population mobility and technology exchange

The Regional Coordinated Development Policy refers to a strategy aimed at promoting balanced economic and social development across regions by fostering cooperation, sharing resources, and reducing disparities. Meanwhile, Institutional Mechanisms for Promoting Social Mobility of Labor and Talent seeks to enhance the attractiveness of small cities and middle-sized cities by modifying settlement policies, improving the allocation of human capital, and fostering a more dynamic and equitable labor market.

In this study, we explore a scenario of inter-city exchange and disparities. Ideally, in HighInter scenario, less developed cities can accelerate their catch-up through more effectively learning in technology, accumulating capital and labor with more balanced labor distribution. In MedInter scenario, we assume moderate technology exchange, and the migration and employment level align with trends observed between 2010 and 2020. The LowInter scenario depicts significant population loss and declining employment rates in underdeveloped cities, and lower technology exchange and convergence process, exacerbating disparities among cities. The scenario assumptions and their parameter settings are listed in Table 2.

**Table 2:** Scenario assumptions of GDP projection.

Scenarios	Assumptions	Description and parameter assumptions
Fertility	HighFer	The HighFer scenario assumes effective fertility policy, with total fertility rate (TFR) increasing to the ideal value of 1.8 by 2050 and remaining constant thereafter^29^.
	MedFer	The MedFer scenario assumes that fertility policy prevents a decline in TFR, but the rate remains constant at 1.3 until 2100^29^.
	LowFer	The LowFer scenario assumes TFR drops to 0.7 by 2050, gradually increasing to 0.9 by 2100^29^.
Technological development	HighTech	The HighTech scenario assumes high technological advancement, with a long-term TFP growth rate of 1.0%^6^, high level of TFP growth with 30% higher convergence rates, and long-term capital output elasticity of 0.4.
	MedTech	The MedTech scenario assumes that frontier long-term TFP growth rate is 0.8%, convergence parameter ranges 0.001–0.005, and long-term output elasticity of capital is stable at the level of 0.35^6^.
	LowTech	The LowTech scenario assumes the frontier long-term TFP growth rate as 0.6%^6^, lower level of technological exchange between cities with 30% lower convergence rates, and transition time ranging 10–70 years. Additionally, the long-term output elasticity of capital is assumed to be stable at 0.3.
Intercity interaction and disparities	HighInter	It is characterized by stable migration consistent with 2010 levels before 2020, then gradually decreasing to zero. The labor force participation rate (LFPR) experiences a steady decline of −0.26% before stabilizing in 2030^32^. Key features include uniform convergence parameters across all city tiers and extended technology transfer windows with transition time ranging 25–65 years, promoting balanced economic development through enhanced knowledge spillovers and reduced regional disparities.
	MedInter	Representing a business-as-usual (BAU) reference case, this scenario assumes intermediate levels of technological exchange between cities, reflected in tier-dependent convergence rates. Non-core cities exhibit 20% lower migration attractiveness compared to core cities^6^. While migration rates increase before 2020, mirroring historical trends, employment levels experience a steady decline. The scenario captures moderate spatial inequality with controlled divergence.
	LowInter	This scenario reflects constrained technological diffusion, shortened technology transfer windows with transition time ranging 15–55 years, and amplified convergence rate disparities across city tiers. Small and medium-sized cities face disproportionately large migration outflows before 2020, while the overall employment rate declines rapidly (−0.51% annually) until stabilization in 2030. The outcome demonstrates sharply widening intercity development gaps, simulating fragmented regional growth patterns.

## Data Records

The dataset includes GDP data for 331 cities in China, with a 27-scenario matrix of GDP values in million yuan based on the 2020 price level, which is available at the public repository Figshare^34^. It is provided in two formats for multidiscipline researchers. First, Excel files contain the GDP data for each administrative unit, stored in “GDP_cityname.csv,” including the 27 scenarios and GDP projections for each city from 2020 to 2100. Second, shapefile maps are named following the convention “TechSSP_InterSSP_FerSSP_Year.shp,” with each shapefile using the EPSG:4326 projection and containing city names and the GDP values for each scenario and year. These files are all accessible via figshare.

**Fig. 2:**
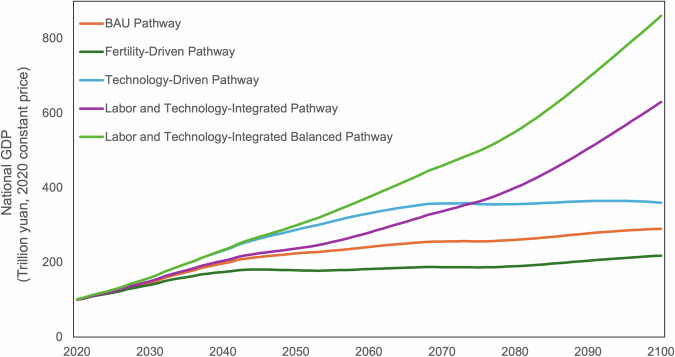
Representative pathways of national GDP from 2020 to 2100.

**Fig. 3:**
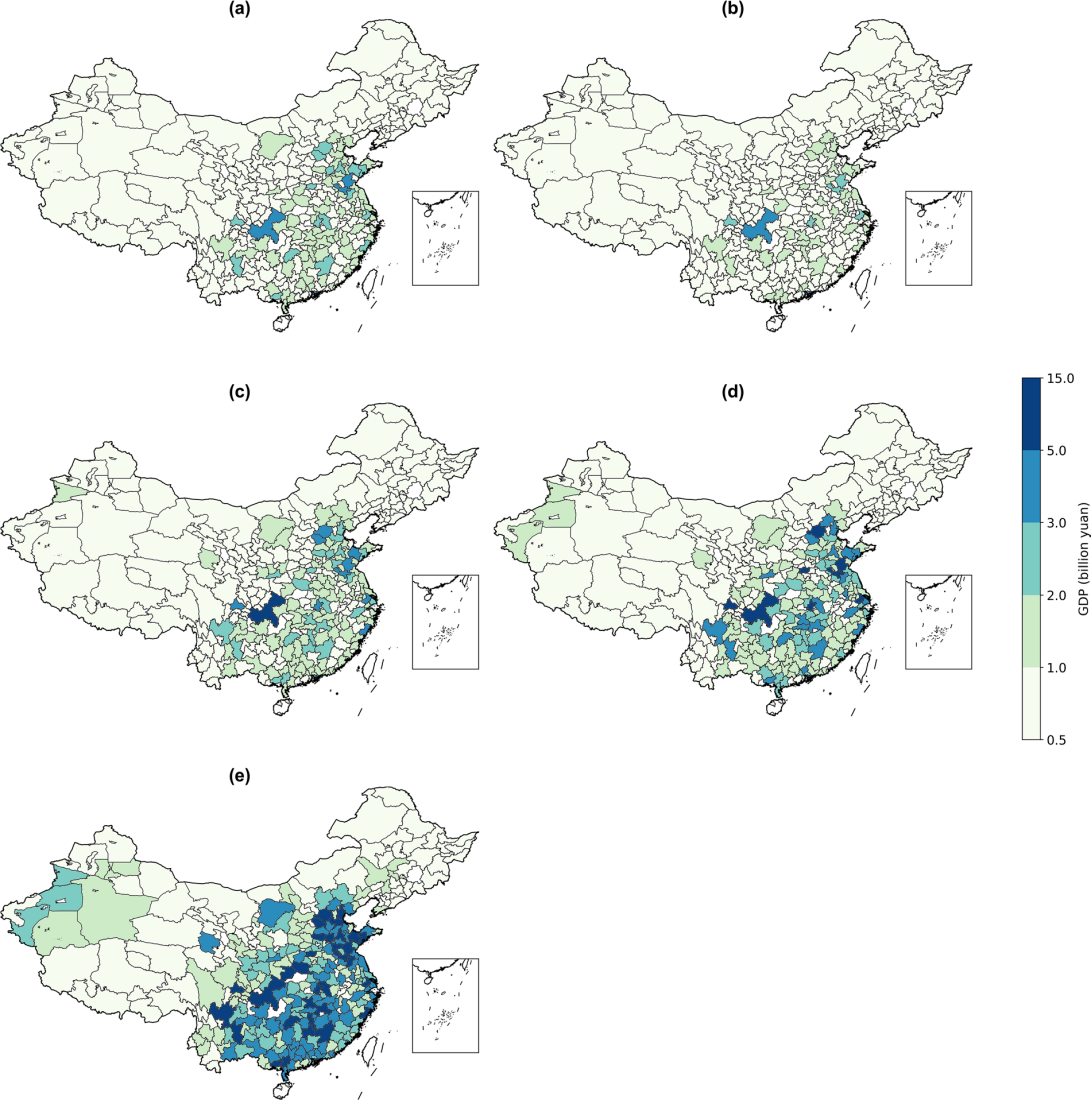
The city-level GDP projection at 2100 under five representative pathways in 2100 (trillion yuan, 2020 price level). (**a–e**) represent (**a**) BAU Pathway (**b**) Technology-Constraint Pathway (**c**) Labor-Constraint Pathway (**d**) Fragmented Regional Pathway (**e**) Balanced Prosperity Pathway respectively.

From the 27 GDP development scenarios, this study identifies five representative pathways to demonstrate possible trends in future GDP growth, as in Table 3. (a) The MedTech-MedFer-MedInter represents the Business-as-Usual (BAU) pathway, where technology development, fertility rates, and inter-city interactions all follow moderate trajectories. (b) LowTech-HighFer-LowInter illustrates Technology-Constraint Pathway, characterized by a demographic shift alongside technological stagnation, compounded by a decline in inter-city cooperation. This could hinder innovation and negatively affect the overall quality of life in cities. (c) HighTech-LowFer-HighInter illustrates Labor-Constraint Pathway. The impact of the two-child and three-child policies may not be significant, but technologies like AI and renewable energy are seeing innovation. At the same time, policies encouraging inter-city collaboration and various city assistance programs are being implemented. (d) HighTech-HighFer-LowInter illustrate Fragmented Regional Pathway, where, despite population growth and technological advancements, cities operate independently, lacking coordination and strategic planning, resulting in disjointed regional development. (e) HighTech-HighFer-HighInter is Balanced Prosperity Pathway. It represents an ideal situation where policies are fully effective, though it may be an unattainable boundary. Figure 2 presents the national GDP projection under five representative pathways. Figure 3 illustrats the city-level GDP in 2100 under 5 representative pathways.

**Table 3:** Characteristics of Representative GDP Pathways.

Pathway Name	Technology	Fertility	Inter-city interactions	Related SSP
Business-as-Usual Pathway	Med	Med	Med	SSP2
Technology-Constraint Pathway	Low	High	Low	—
Labor-Constraint Pathway	High	Low	High	SSP1
Fragmented Regional Pathway	High	High	Low	—
Balanced Prosperity Pathway	High	High	High	—

## Technical Validation

### Error analysis

The projected city-level GDP is based on the historical value of 2020. Among the various scenarios, the MedTech-MedFer-MedInter scenario represents the business-as-usual (BAU), which is used for the technical validation compared with actual trend of GDP growth. To evaluate the predictive accuracy and bias in the GDP projection, we employ two widely used accuracy indicators in population projections: the absolute percentage error (APE) and the algebraic percentage error (PE). The calculation methods are described in Eqs. (12, 13).

12 $${\rm{APE}}( \% )=\left|\frac{{{\rm{P}}}_{{\rm{t}}}-{{\rm{A}}}_{{\rm{t}}}}{{{\rm{A}}}_{{\rm{t}}}}\right|\times 100 \% $$

13 $${\rm{PE}}( \% )=\left(\frac{{{\rm{P}}}_{{\rm{t}}}-{{\rm{A}}}_{{\rm{t}}}}{{{\rm{A}}}_{{\rm{t}}}}\right)\times 100 \% $$

Where, P_t_ represents the projected result and A_t_ represents the corresponding actual value. Therefore, projection is overestimated when PE is positive, and vice versa. City-level GDP from 2020 to 2023 for the 31 provinces of China are collected from provincial statistical yearbooks to verify the estimated results from the Cobb-Douglas production function and determine the applicability of the economic projection model in China. In addition, consumer price index are collected from the Bureau of Statistics of China to standardize the GDP data in the different years.

Firstly, we compare the national GDP projection with statistical value, it turns out that the algebraic percentage error in 2020–2023 is ranged from 1.53%–2.83%. We also conducted a retrospective validation by applying the same recursive equations with different base years (2010, 2015, and 2020) to simulate GDP. As shown in Table 4, The resulting algebraic percentage errors were 2.77%, 5.13%, and 4.08% for the base years 2020, 2015, and 2010, respectively. The results confirm that while the error margin increases with longer time spans, the model maintains reasonable reliability.

**Table 4:** Percentage error of national GDP projection in 2020–2023.

Year		2020	2021	2022	2023
Real GDP	Trillion yuan (2020 constant value)	101.36	109.57	112.85	118.72
GDP estimates based on 2020	Trillion yuan (2020 constant value)	99.80	104.54	110.46	115.36
	Percentage error %	−1.53	−4.59	−2.12	−2.83
GDP estimates based on 2015	Trillion yuan (2020 constant value)	97.53	102.04	107.72	112.41
	Percentage error %	−3.78	−6.87	−4.55	−5.32
GDP estimates based on 2010	Trillion yuan (2020 constant value)	99.88	103.58	108.42	112.21
	Percentage error %	−1.46	−5.46	−3.93	−5.48

**Fig. 4:**
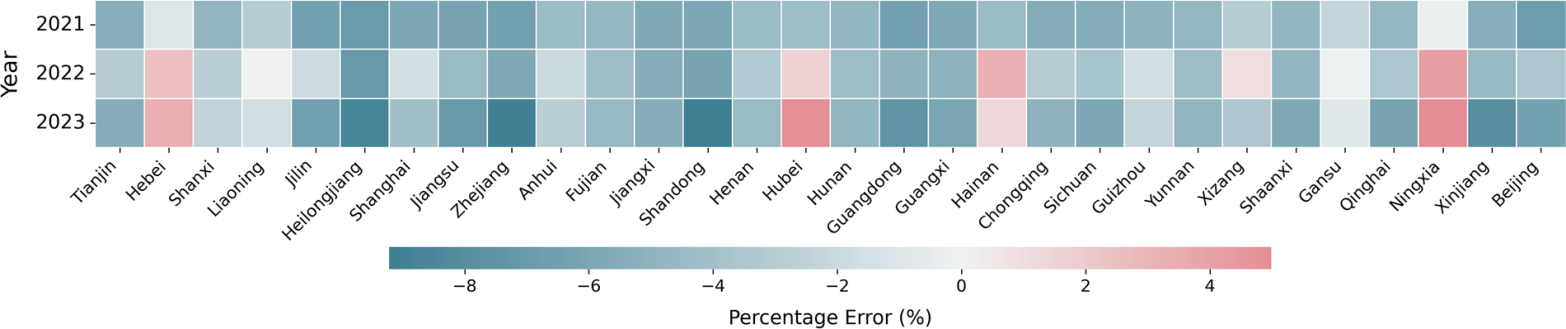
Percentage errors of provincial-level GDP projection in 2021–2023.

We analysis the estimation errors at provincial level, showing our examination of provincial GDP estimation with an average underestimation of −4.83% in 2021, −2.63% in 2022, and −4.13% in 2023. Notably, the variability of errors increased significantly over this period, with the standard deviation rising from 1.59 in 2021 to 3.75 in 2023, suggesting growing disparities in estimation accuracy across provinces. When it comes to city-level GDP estimation errors, percentage errors (PE) shows −4.11% in 2021, −1.73% in 2022, −2.48% in 2023, respectively. Notably, error variability increased substantially over this period, with the standard deviation growing from 2.07% to 3.94%, indicating widening disparities in estimation accuracy across cities. The provincial level percentage errors are shown in Fig. 4.

**Fig. 5:**
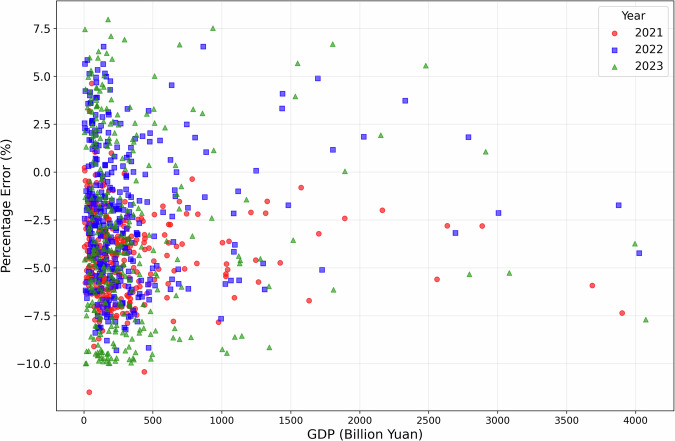
Percentage errors of city-level GDP projection in 2021–2023.

**Fig. 6:**
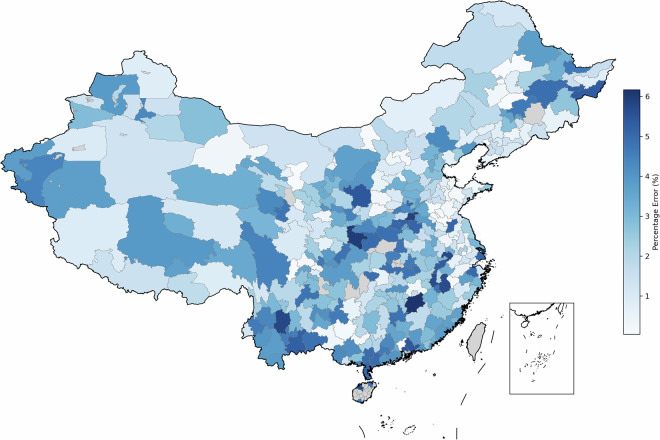
Average percentage errors of city-level GDP projection in 2021–2023.

At the national level, the majority of cities (259, representing 78.2% of the total) maintain relatively accurate estimates with absolute percentage errors (APE) below 5%, collectively accounting for 83.1% of China’s total GDP. Regional disparities are particularly noteworthy, with high-error cities (APE > 6%) predominantly clustered in western and southern regions - including Yingtan (6.91%), Ngari (6.89%), and Qinzhou (6.87%) - while eastern coastal cities demonstrate notably higher accuracy, exemplified by Weihai (0.005%) and Xiamen (0.20%). These geographical variations suggest that less developed areas tend to show systematically higher errors, potentially reflecting differences in statistical capacity or economic structure. When examining cities by economic tiers, we find that Tier 1 cities (top 20% by GDP) exhibit slightly better estimation accuracy (mean percentage error = −2.11%) compared to Tier 2 (−2.97%) and Tier 3 cities (−2.85%), though these inter-tier differences are less pronounced than the observed regional disparities. The city-level percentage errors are shown in Figs. 5 and 6.

### SSPs comparision

We also compared the GDP estimates with the results from IPCC SSPs Database considering the assumptions of fertility, migration and TFP growth^35^. Latest economic projections of the International Institute for Applied Systems Analysis (IIASA), Potsdam Institute for Climate Impact Research (PIK), and Organization for Economic Cooperation and Development (OECD) are collected^36^. For consistent comparison with OECD, PIK, and IIASA projections, we implemented three key adjustments: (1) applied PPP conversion factors to standardize all values to RMB units^37^, (2) used China’s 2020 CPI to anchor projections to constant price levels. Long-term validation confirms our projections converge with benchmark studies by 2100. The PPP conversion factor is retrieved from the World Bank database. Consumer price index (CPI) data are collected from the Bureau of Statistics of China to standardize the GDP value in different years.

**Fig. 7:**
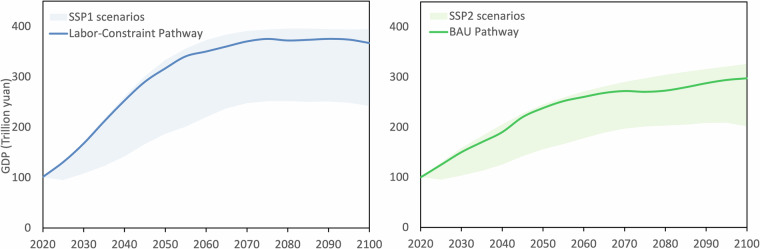
Comparison of GDP projections under SSP1 and SSP2 scenarios. (**a**) compares the GDP estimates of Labor-Constraint Pathway from this study (solid line) alongside the SSP1 projections from OECD, PIK, and IIASA (shaded region). (**b**) compares the GDP estimates of BAU Pathway from this study (solid line) alongside the SSP2 projections from OECD, PIK, and IIASA (shaded region).

We selected the HighTech-LowFer-HighInter (c. Labor-Constraint Pathway) and MedTech-MedFer-MedInter (a. BAU Pathway) for comparison, as they closely align with the SSP1 and SSP2 pathways, respectively: SSP1 features low fertility, medium migration, medium-high frontier TFP growth, and high-speed convergence, while SSP2 refers to medium fertility, medium migration, medium frontier TFP growth, and medium-speed convergence. Figure 7 presents the comparison results of our estimates and other SSPs projections. Under the SSP1 scenario, our study estimates GDP at 337 trillion yuan, compared to 343 trillion yuan by OECD, 394 trillion yuan by PIK and 242 trillion yuan by IIASA; under the SSP2 scenario, our estimate is 247 trillion yuan, while OECD, PIK and IIASA project 311, 326 and 202 trillion yuan, respectively. While the other SSPs, particularly inequality SSP4 and fossil-fuel-dominant SSP5^8,9^, is not applicable in China due to recent effort on comprehensive poverty reduction, and carbon neutrality, without suitable parameter estimates in the datasets.

### Sensitivity analysis

**Fig. 8:**
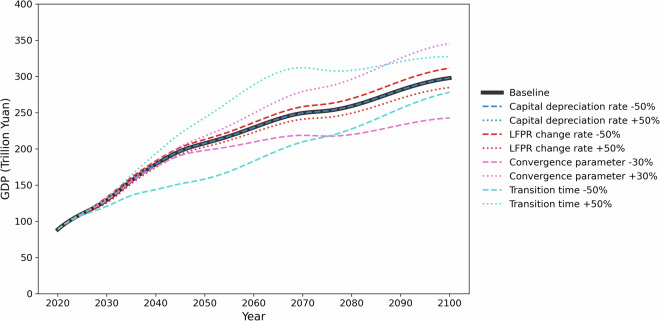
Sensitivity of China’s national GDP to key parameters under the BAU pathway.

Within this sensitivity analysis, robustness is demonstrated by moderate sensitivities to key parameters. Sensitivity is checked against four major parameters: LFPR change rate, which affects the future labor force estimates, capital depreciation *δ*, which affects the initial physical capital investment, convergence parameter β and transition time *τ*, which dicide the future TFP growth. The sensitivity analysis is shown in Fig. 8. We focus the discussion of the BAU pathway sensitivity at the national level.

The convergence parameter emerges as the most influential factor, variations of ±30% in this parameter generate asymmetric effects ranging from −18.5% to +16% in GDP outcomes, with slower convergence rates particularly constraining long-term growth potential. The transition time parameter exhibits moderate but persistent effects on GDP growth trajectories. When subjected to 50% variations, this parameter produces GDP impacts ranging from −6.6% to +10% by 2100, primarily by altering the dynamics of convergence toward the technological frontier. Labor force participation changes demonstrate stable linear responses, with 50% variations translating to consistent ±4.5% GDP impacts, confirming established theoretical expectations about labor’s role in growth models. Capital depreciation variations show complete neutrality in these sensitivity tests. This parameter hierarchy suggests that interventions targeting technological diffusion and institutional transitions will likely yield greater GDP impacts than those focused exclusively on factor accumulation.

### Uncertainty analysis

Our city-level GDP projections, while carefully constructed using the Cobb-Douglas production model and accounting for China’s unique socio-economic conditions, are subject to several sources of uncertainty. These uncertainties arise from data limitations, modeling assumptions, and external factors that are difficult to quantify over an 80-year projection horizon. Below, we outline the key uncertainties and their potential implications.

A significant challenge in city-level GDP modeling is the lack of high-precision data for certain key inputs. Some of variables are only available at the provincial level, such as price index of fixed capital investment, education levels and age structure. For certain cities, historical fixed capital investment data are missing, particularly before 1990. While we apply scaling factors to maintain consistency, these approximations may not fully capture city-specific dynamics. These data constraints mean that our projections may not fully reflect local economic idiosyncrasies, particularly for smaller or less economically developed cities.

While our model incorporates technology, fertility, and intercity interactions, other critical factors influencing GDP—such as environmental shocks and resource endowments—are not explicitly modeled due to methodological and data constraints. Although climate-related disruptions can have severe economic consequences, their short-term and stochastic nature makes them difficult to integrate into long-term projections. We acknowledge this limitation and emphasize that our results should be interpreted as conditional on the absence of major climate-induced economic shocks. While national models (e.g., ENV-Linkages) incorporate resource constraints, city-level data on subsoil assets, sectoral energy consumption, and resource-specific capital stocks are largely unavailable. This omission may lead to over- or underestimation of GDP in resource-dependent cities.

Future policy shifts, economic disruptions, and unforeseen demographic changes could significantly alter GDP trajectories. Our scenarios assume continuity in key policies (e.g., two-/three-child policies, regional development strategies), but abrupt changes—such as new migration restrictions, trade barriers, or technological stagnation—could invalidate some projections. Additionally, while we model heterogeneous city-level convergence, structural economic transformations like shifts from manufacturing to services may occur at different speeds than anticipated.

Our model designates Shenzhen as the national total factor productivity (TFP) leader due to its empirically verified high productivity. However, this assumption may introduce biases because industrial structure mismatch: Shenzhen’s ICT-dominated economy differs from resource-heavy or manufacturing-centric cities, potentially skewing convergence dynamics. Other cities (e.g., Beijing for R&D-intensive sectors or Shanghai for finance) might serve as better productivity benchmarks for certain regions. To mitigate this, we incorporate differentiated convergence speeds based on city type, but future work could explore multi-leader or sector-specific TFP benchmarks.

## Usage Notes

The city-level GDP projections provided in this dataset are designed to support interdisciplinary research and policy analysis, particularly in climate impact assessments, regional economic planning, and sustainable development studies. Researchers can leverage these projections to evaluate socioeconomic drivers under divergent scenarios or integrate them with environmental models (e.g., emission inventories, energy systems) where subnational economic activity is a critical input. For climate-related studies, the SSP1-aligned Labor-Constraint Pathway offers a low-fertility/high-productivity reference, while the BAU (SSP2) scenario reflects moderate trends consistent with China’s recent development trajectory.

Users should note that projections for smaller cities may exhibit higher uncertainty due to historical data limitations in capital stock and labor statistics; sensitivity analysis is recommended where precision is essential. To ensure compatibility with broader frameworks, the dataset excludes SSP4, SSP5 scenarios inconsistent with China’s policy direction. Annual GDP values are provided in both absolute and indexed (2020 baseline) formats to facilitate cross-scenario comparisons. For dynamic modeling applications, supplementary data on intercity interactions (e.g., technology diffusion, population flows) may be incorporated, as detailed in the Technical Validation section.

## Data Availability

The datasets^34^ supporting this study are available in three categories: (1) Geospatial data comprising “GDP_Shp_Year”, containing city-level GDP distributions across 5 representative scenarios in Shapefile format with WGS84/EPSG:4326; (2) Tabular data including “GDP_administration” and “TFP_administration” with economic indicators (2020 constant million yuan) in CSV format; and (3) Computational materials in “Python_Code” containing processing scripts and raw inputs. All files use standardized naming conventions and maintain consistent directory structures.

## Code availability

All the codes for creating nationwide, provincial and city-level GDP datasets are written with Python version 3.12.4, which are stored in public repository Figshare^34^. Explanations are internalized in the script to help users with implementation.
